# DRD2 Agonist Cabergoline Abolished the Escape Mechanism Induced by mTOR Inhibitor Everolimus in Tumoral Pituitary Cells

**DOI:** 10.3389/fendo.2022.867822

**Published:** 2022-06-03

**Authors:** Federica Mangili, Emanuela Esposito, Donatella Treppiedi, Rosa Catalano, Giusy Marra, Genesio Di Muro, Anna Maria Barbieri, Marco Locatelli, Andrea G. Lania, Alessandra Mangone, Anna Spada, Maura Arosio, Erika Peverelli, Giovanna Mantovani

**Affiliations:** ^1^ Department of Clinical Sciences and Community Health, University of Milan, Milan, Italy; ^2^ Neurosurgery Unit, Fondazione Istituto di Ricovero e Cura a Carattere Scientifico (IRCCS) Ca’ Granda Ospedale Maggiore Policlinico, Milan, Italy; ^3^ Department of Pathophysiology and Transplantation, University of Milan, Milan, Italy; ^4^ Department of Biomedical Sciences, Humanitas University, Pieve Emanuele, Italy; ^5^ Endocrinology and Diabetology Unit, Istituto di Ricovero e Cura a Carattere Scientifico (IRCCS) Humanitas Research Hospital, Rozzano, Italy; ^6^ Endocrinology Unit, Fondazione Istituto di Ricovero e Cura a Carattere Scientifico (IRCCS) Ca’ Granda Ospedale Maggiore Policlinico, Milan, Italy

**Keywords:** pituitary neuroendocrine tumors, mTOR inhibitors, everolimus, AKT phosphorylation, dopamine receptor type 2, cabergoline

## Abstract

The mammalian target of rapamycin (mTOR) inhibitor everolimus has been shown to display antiproliferative effects on a wide spectrum of tumors. *In vitro* studies demonstrated that everolimus inhibited pituitary neuroendocrine tumor (PitNET) cell growth in a subset of patients. Sensitivity to everolimus is reduced by an escape mechanism that increases AKT phosphorylation (p-AKT), leading to pro-survival pathway activation. Dopamine receptor type 2 (DRD2) mediates a reduction of p-AKT in a subgroup of non-functioning PitNETs (NF-PitNETs) and in prolactin-secreting tumor cells (MMQ cells) through a β-arrestin 2-dependent mechanism. The aim of this study was to investigate the efficacy of everolimus combined with DRD2 agonist cabergoline in reducing NF-PitNET primary cells and MMQ cell proliferation and to evaluate AKT phosphorylation and a possible role of β-arrestin 2. We found that 9 out of 14 NF-PitNETs were resistant to everolimus, but the combined treatment with cabergoline inhibited cell proliferation in 7 out of 9 tumors (-31.4 ± 9.9%, *p* < 0.001 *vs*. basal) and reduced cyclin D3 expression. In the everolimus-unresponsive NF-PitNET group, everolimus determined a significant increase of p-AKT/total-AKT ratio (2.1-fold, *p* < 0.01, *vs*. basal) that was reverted by cabergoline cotreatment. To investigate the molecular mechanism involved, we used MMQ cells as a model of everolimus escape mechanism. Indeed everolimus did not affect MMQ cell proliferation and increased the p-AKT/total-AKT ratio (+1.53 ± 0.24-fold, *p* < 0.001 *vs*. basal), whereas cabergoline significantly reduced cell proliferation (-22.8 ± 6.8%, *p* < 0.001 *vs*. basal) and p-AKT. The combined treatment of everolimus and cabergoline induced a reduction of both cell proliferation (-34.8 ± 18%, *p* < 0.001 *vs*. basal and *p* < 0.05 *vs*. cabergoline alone) and p-AKT/total-AKT ratio (-34.5 ± 14%, *p* < 0.001 *vs*. basal and *p* < 0.05 *vs*. cabergoline alone). To test β-arrestin 2 involvement, silencing experiments were performed in MMQ cells. Our data showed that the lack of β-arrestin 2 prevented the everolimus and cabergoline cotreatment inhibitory effects on both p-AKT and cell proliferation. In conclusion, this study revealed that cabergoline might overcome the everolimus escape mechanism in NF-PitNETs and tumoral lactotrophs by inhibiting upstream AKT activation. The co-administration of cabergoline might improve mTOR inhibitor antitumoral activity, paving the way for a potential combined therapy in β-arrestin 2-expressing NF-PitNETs or other PitNETs resistant to conventional treatments.

## Introduction

In recent years, PI3K–AKT–mTOR inhibitors have been proposed as an alternative therapeutic strategy for tumors resistant to conventional drugs. Indeed mTOR inhibitors, like everolimus, torin-1, or rapamycin, displayed successful results in a wide spectrum of tumors. Specifically, everolimus showed a promising perspective in tumors such as HER2/neu negative advanced breast cancer, bronchial and renal cell carcinoma, unresectable or metastatic neuroendocrine pancreatic tumors, or nonfunctional gastrointestinal and lung-originating ones (1).

The PI3K–AKT–mTOR pathway is known to have a fundamental role in regulating cell proliferation, survival, and metabolism. In particular, this pathway has been found overactivated in pituitary neuroendocrine tumors (PitNETs) compared with normal pituitary (2–4), supporting its contribution to PitNET progression (5).

Non-functioning (NF)-PitNETs cause visual field deficits and neurologic manifestations due to mass spread effects, and even if generally benign, they may invade surrounding structures and present resistance to medical treatments. Surgical tumor debulking is currently considered the first-line approach, and to date, NF-PitNETs are still orphans of effective medical therapy (6, 7).

*In vitro* studies displayed that mTOR inhibitors induced a reduction of cell growth and viability in primary cultured NF-PitNET cells (8–11). On the other hand, several different mechanisms have been demonstrated in literature to explain how sensitivity to mTOR inhibitors could be reduced in different kinds of tumors (12).

Among these, one of the most outlined is an escape mechanism that induces an upstream increase of AKT phosphorylation levels (p-AKT) through insulin receptor substrate-1 (IRS-1) triggering, leading to pro-survival pathway activation (13). Indeed the PI3K–AKT–mTOR pathway activation results in a negative feedback loop, mediated by the mTOR/S6K-dependent loss of IRS-1 expression (14). This mechanism of negative feedback is lost when mTOR activity is inhibited with everolimus, flowing into an increase of p-AKT levels and thereby in an enhancement of cell proliferation (13, 15).

The inhibition of mTORC1 has been reported to induce AKT activation in numerous cell types (16–18), unveiling the fact that mTOR inhibitors block mTORC1 activity but do not alter mTORC2 assembly, accordingly with the direct phosphorylation of AKT at Ser 473 (19). In this regard, an increase of AKT activity has been shown to be one of the major contributors that diminished mTOR inhibitors’ anticancer activity (20).

This limited efficacy of everolimus due to p-AKT re-activation could be overcome by upstream AKT phosphorylation inhibition.

In this connection, a new molecular mechanism that contributes to confer sensitivity or resistance to dopamine agonists (DAs) in PitNETs was recently revealed (21). More specifically, it was demonstrated that, in a subset of NF-PitNETs and in rat prolactin (PRL)-secreting tumoral cells (MMQ), the treatment with a DRD2-selective agonist (BIM53097) leads to p-AKT inhibition, flowing into a reduction of cell proliferation through a β-arrestin 2-dependent mechanism. Briefly, in this work, it was demonstrated that β-arrestin 2 transfection in NF-PitNETs lacking its expression restored the ability of the dopamine agonist to exert its antimitotic action (21). Moreover, in MMQ cells silenced for β-arrestin 2, an opposite effect of BIM53097 on p-AKT was observed, together with a complete loss of its antiproliferative activity. These data unveiled a new molecular mechanism that contributes to confer sensitivity or resistance to DAs in pituitary tumors. In tumoral lactotrophs and NF-PitNETs, the lack of β-arrestin 2 prevents the inhibitory effect of DRD2 on AKT pathway activation with a consequent resistance to the antimitotic action of DAs.

Based on the above-mentioned premises, the aim of this study was to test the efficacy of cabergoline in increasing the sensitivity of human primary cultured NF-PitNET cells to the antiproliferative effects of everolimus and to investigate the contribution of β-arrestin 2.

## Materials and Methods

### Cell Cultures

The local ethics committee previously approved the study, and each patient involved gave informed consent to the use of his/her tumor sample. A brief description of the patient and tumor characteristics is reported in **Table 1**. Human pituitary cells were obtained by trans-sphenoidal surgery from patients with NF-PitNET. The tissues were partially frozen for subsequent molecular analysis and partially enzymatically dissociated as previously described (22). The dispersed cells were cultured in Dulbecco’s modified Eagle’s medium supplemented with 10% fetal bovine serum (FBS), 2 mM glutamine, and antibiotics (Gibco, Invitrogen, Life Technologies Inc., Carlsbad, CA, USA). Rat tumoral pituitary MMQ cells (ATCC CRL-10609) were grown in RPMI medium (Life Technologies, Thermo Fisher, Carlsbad, CA, USA) supplemented with 15% horse serum, 2.5% FBS, 2 mM glutamine, and antibiotics.

**Table 1:** Radiological pre- and post-surgery information of NF-PitNET patient’s derived samples.

NF-PitNET samples	Gender	Age at surgery (years)	Radiology information			Pre-surgery information			Post-surgery information	

			Macroadenoma	Tumor’s maximum dimension (mm)	Cavernous sinus invasion	Pre-surgery therapy	Hypopituitarism	Number of deficits	Hypopituitarism	Number of deficits
1	F	44	Yes	27.5	Yes	No	Yes	1	Yes	2
2	F	74	Yes	23	Yes	No	Yes	1	No	0
3	F	35	Yes	33	Yes	No	Yes	1	Yes	1
4	F	54	Yes	19	Yes	No	Yes	2	Yes	2
5	F	72	Yes	25	Yes	No	No	0	No	0
6	F	54	Yes	26	Yes	No	No	0	Yes	3
7	M	83	Yes	30	Yes	No	Yes	1	No	0
8	F	43	Yes	27	No	No	No	0	No	0
9	M	72	Yes	26	No	Yes^a^	Yes	3	Yes	3
10	M	53	Yes	26	Yes	No	Yes	1	Yes	1
11	M	68	Yes	27	Yes	No	Yes	1	No	0
12	F	62	Yes	24	Yes	No	No	0	Yes	1
13	M	68	Yes	25	Yes	No	Yes	3	Yes	4
14	M	68	Yes	14	No	No	No	0	No	0

a Cabergoline, 1 mg/week for 2 years. A summary of the characteristics of the patients from whom the samples are derived is shown. The gender and age at surgery of the patients are shown, together with the radiological characteristics of the tumor (presence of macroadenomas, its maximum dimension, and if cavernous sinus was invaded). Details about the presence of hypopituitarism and deficits before and after the surgery are reported

### Chemicals

Everolimus and cabergoline were both purchased from Sigma-Aldrich. The powders were suspended in sterile dimethyl sulfoxide, stocked at -80°C, and diluted immediately before use in complete culture medium.

### Proliferation Assay

Cell proliferation was determined by the colorimetric measurement of 5-bromo-2′-deoxyuridine (BrdU) incorporation during DNA synthesis in proliferating cells as previously described (23). MMQ and NF-PitNET cells were seeded in starved medium in a 96-well polylysine-coated plate at a density of 2 or 5 × 10^4^ cells/well, respectively. The cells were incubated for 72 h in complete medium with cabergoline, everolimus, or their combination. BrdU incorporation in newly synthesized DNA was then allowed at 37°C for 24 h (NF-PitNETs) or 2 h (MMQ). Therefore, the assay was performed in accordance with the instruction of the manufacturer (Cytiva, Life Science, Marlborough, MA, USA).

### Western Blot Analysis

MMQ and NF-PitNETs cells were seeded at a density of 3 × 10^5^ cells/well in a 6-well plate for western blot analysis. After 24 h, the cells were treated with cabergoline and everolimus, alone or in combination, for 3 h at 37°C. Total proteins were quantified by bicinchoninic acid assay; 60 µg were separated on SDS/polyacrylamide gels and transferred to a nitrocellulose filter. Total-AKT and cyclin D3 antibodies were used at 1:1,000, phospho-AKT (Ser473) and β-arrestin 2 antibodies at 1:2,000 (Cell Signaling, Danvers, MA, USA). Primary antibodies were incubated overnight at 4°C; secondary antibodies anti-rabbit or anti-mouse (Cell Signaling, Danvers, MA, USA) was used at 1:2,000 at room temperature for 1 h. Anti-GAPDH antibody (Invitrogen, Thermo Fisher, CA, USA) were used at 1:4,000 for 1 h at room temperature. Chemiluminescence was detected using ChemiDOC-IT Imaging System (UVP, Upland, CA, USA) and analyzed with NIH ImageJ software. β-arrestin 2 expression level analysis was performed on frozen samples of human NF-PitNETs.

### β-Arrestin 2 Silencing

MMQ cells were silenced with rat β-arrestin 2 smart pool siRNAs and Dharmafect transfection agent 4 (Dharmacon, GE Healthcare Life Sciences, Chicago, IL, USA) as previously described (21). In each experiment, a negative control siRNA (non-targeting sequence without a significant homology to the sequence of human, mouse, or rat transcripts) was used. For each experiment, the silencing efficiency was tested by western blot analysis, and only experiments with at least 70% silencing efficiency were considered.

### Transcription Factor Analysis

Total RNA was extracted from human NF-PitNET frozen tissues conserved at -80°C with Trizol Reagent (Life Technologies, Carlsbad, CA, USA) using standard methods. Moreover, 2 μg of total RNA were reverse-transcribed with RevertAid H Minus First Strand cDNA Synthesis Kit (Thermo Fisher Scientific, Whaltam, MA, USA). Then, 1 μl of cDNA was used for PCR with a specific primer amplifying human SF-1 (Fw: 5′-AGCTGCAAGGGCTTCTTCAA-3′; Rw: 5′-GAATCTGTGCCTTCTTCTGC-3′) and DRD2 (Fw: 5′-AGACCAGAACGAGTGCATCA-3′; Rw: 5′-CGCCAAACCAGAGAAGAATG-3′). A GAPDH transcript was used as the housekeeping standard.

### Statistical Analysis

The results are expressed as mean ± SD. A paired two-tailed Student’s *t*-test was used to detect the significance between two series of data. Two-way ANOVA test was used to analyze the significance between two different groups of data. In addition, *p <*0.05 was accepted as statistically significant.

## Results

### *In Vitro* Responsiveness of NF-PitNET Primary Cultures to Cabergoline, Everolimus, and Their Cotreatment

**Figure 1:**
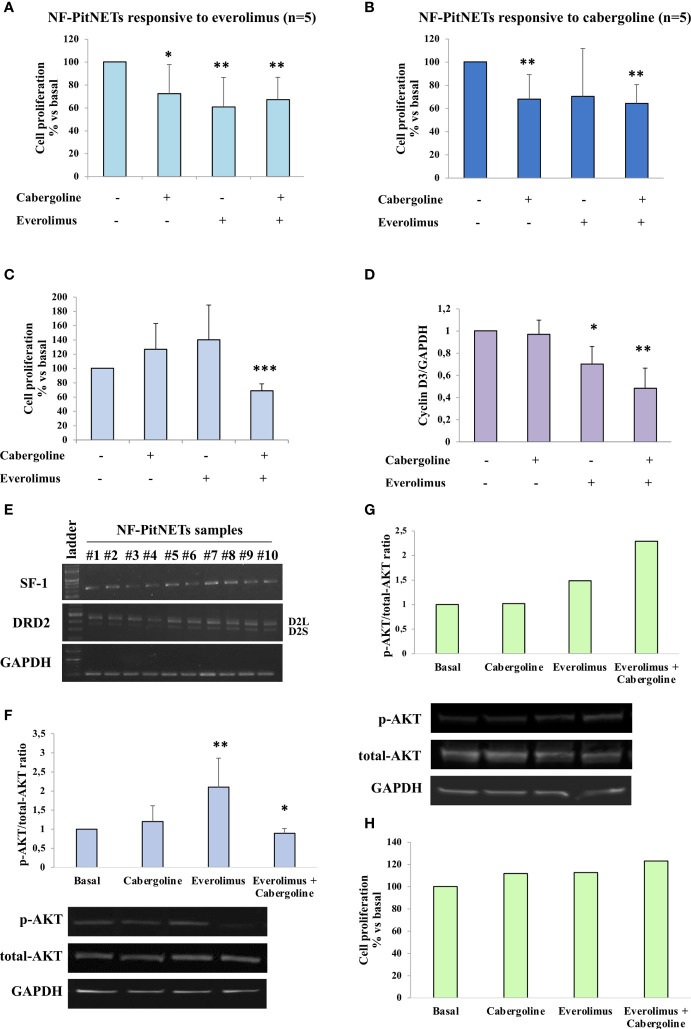
NF-PitNET cell primary culture response to everolimus, cabergoline, and cotreatment on AKT phosphorylation and cell proliferation. Primary cultured of NF-PitNET cells **(A–H)** were treated with 100 nM cabergoline and 1 nM everolimus for 72 h **(A–C**, **H)** or 3 h **(D, F, G)** at 37°C, administered singularly or in combination. **(A–C)** BrdU incorporation in newly synthesized DNA was measured. Data represent the mean ± SD normalized *vs*. respective basal of 14 different NF-PitNETs primary cultures: *n* = 5 responsive to everolimus **(A)**, *n* = 5 responsive to cabergoline **(B)**, *n* = 7 resistant to both drugs but responsive to the cotreatment **(C)**. Each determination was done in triplicate. *p < 0.05, **p < 0.01, ***p < 0.001 *vs*. corresponding basal. **(D)** The graph shows the quantification of cyclin D3 normalized to GAPDH (mean value ± SD from 3 primary cultures of NF-PitNETs cells). *p < 0.05, **p < 0.01 *vs*. corresponding basal. **(E)** RT-PCR analysis of NF-PitNET samples (*n* = 10) in order to detect SF-1, D2L, and D2S DRD2 isoform expression. GAPDH expression was analyzed as control. Representative images are shown. **(F)** The graph shows the quantification of p-AKT/total AKT normalized to the basal. Data represent mean ± SD of 6 different NF-PitNET samples. Representative immunoblots are shown. *p < 0.05; **p < 0.01, *vs*. corresponding basal. **(G, H)** NF-PitNET primary cultured cells resistant to cabergoline, everolimus, and cotreatment were analyzed. **(G)** The graph shows the quantification of p-AKT/total AKT normalized to the basal and representative immunoblots. **(H)** Measurement of BrdU incorporation in newly synthesized DNA.

The everolimus treatment was effective in reducing cell proliferation in 5 out of 14 NF-PitNET primary cultured cells (-39.2 ± 25.8% at 1 nM, *p* < 0.01 *vs*. basal) (**Figure 1A**). An everolimus dose was chosen based on literature data (8), and preliminary dose–response experiments were performed in NF-PitNET cultured cells (**Supplementary Figure S1A**). In unresponsive tumors, no reduction of cell proliferation was observed even at higher doses of everolimus (10 nM, 100 nM, and 1 µM) (**Supplementary Figure S1B**). Cabergoline incubation determined a reduction of NF-PitNET primary cell proliferation in 5 out of 14 samples (-32 ± 21.2% at 100 nM, *p* < 0.01 *vs*. basal, **Figure 1B**). In total, 4 out of these 5 tumors were responsive to both drugs. In responsive tumors, the cotreatment of everolimus and cabergoline did not enhance the efficacy of the drugs administered singularly (**Figures 1A, B** **)**. Moreover, 8 out of 14 NF-PitNETs were resistant to both cabergoline and everolimus (**Table 2**).

**Table 2:** NF-PitNETs primary cultured responsiveness to everolimus, cabergoline, and their cotreatment.

NF-PitNET			
sample	Cabergoline	Everolimus	Cabergoline + everolimus
1			
2			
3			
4			
5			
6			
7			
8			
9			
10			
11			
12			
13			
14			

a Primary cultures (*n* = 14) of NF-PitNET cells were treated with 100 nM cabergoline and 1 nM everolimus for 72 h at 37°C, administered singularly or in combination, and BrdU incorporation in newly synthesized DNA was measured. The table presents the responsiveness, in terms of proliferation reduction, of each sample to each treatment. Responsive NF-PitNETs are indicated in green color and the unresponsive ones in red.

Interestingly, in NF-PitNETs resistant to everolimus, the coadministration of cabergoline was effective in inhibiting cell proliferation in 7 out of 9 tumors (-31.4 ± 9.9%, *p* < 0.001 *vs*. basal) (**Figure 1C** and **Table 2**). Similarly, the combined treatment exerted a strong reduction of cyclin D3 expression (-52 ± 18%, mean of 3 different tumors, *p* < 0.01 *vs*. basal, **Figure 1D**).

In order to establish if NF-PitNET lineage derivation might affect the responsiveness to cabergoline, everolimus, or both, the expression of transcription factor steroidogenic factor-1 (SF-1), marker of a gonadotrophic lineage (24), was evaluated by RT-PCR analysis. Our results showed a positive expression of SF-1 in all tumor samples (**Figure 1E**). Moreover, all tumors expressed both the long (D2L) and short (D2S) isoforms of DRD2 (**Figure 1E**).

### Everolimus and Cabergoline Effects on AKT Phosphorylation in NF-PitNETs

We then evaluated the AKT activity, testing its phosphorylation status on Ser473 (p-AKT). Our data unveiled that in 6 out of 7 everolimus-unresponsive NF-PitNET group, the 3h everolimus treatment determined a significant increase of the p-AKT/total-AKT ratio (2.1-fold, *p* < 0.01 *vs*. basal), and this effect was reverted by cabergoline cotreatment (**Figure 1F**).

Interestingly, cabergoline was unable to revert the increase of AKT phosphorylation in one everolimus-resistant sample that was also resistant to the antiproliferative effects of the everolimus-and-cabergoline combined treatment, suggesting that the ability to reduce everolimus-dependent AKT phosphorylation is required for the antiproliferative effect (**Figures 1G, H** **)**.

### Cabergoline and Everolimus Cotreatment Reduced Cell Proliferation and AKT Phosphorylation in Everolimus-Resistant MMQ Cells

**Figure 2:**
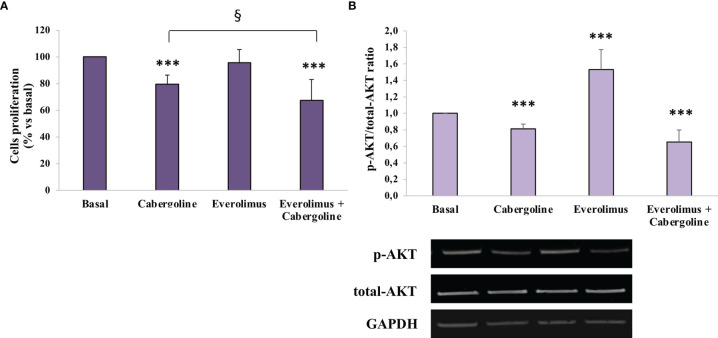
Cabergoline and everolimus cotreatment reduced both cell proliferation and AKT phosphorylation in MMQ cells. MMQ cells were cultured with 100 nM cabergoline and 1 nM everolimus for 72 h **(A)** or 3 h **(B)**, administered singularly or in combination. **(A)** BrdU incorporation in newly synthesized DNA was measured. The experiments were repeated 4 times, and each determination was done in quadruplicate. Values represent mean (± SD) normalized *vs*. respective basal. *** *p* < 0.001 *vs*. corresponding basal; ^§^ *p* < 0.05 *vs*. cabergoline administered alone. **(B)** The graph shows the quantification of p-AKT on Ser473 normalized to total AKT (mean value ± SD from 4 independent experiments). Representative immunoblots are shown. *** *p* < 0.001 *vs*. corresponding basal.

In MMQ cells, everolimus administration did not affect cell proliferation in a range of doses from 0.1 to 100 nM (**Supplementary Figure S1C**), while cabergoline inhibited cell growth (-22.8 ± 6.8%, *p* < 0.001 *vs*. basal), and a greater inhibition was reached after cabergoline and everolimus coincubation (-34.8 ± 18%, *p* < 0.001 *vs*. basal and *p* < 0.05 *vs*. cabergoline) (**Figure 2A**).

**Figure 3:**
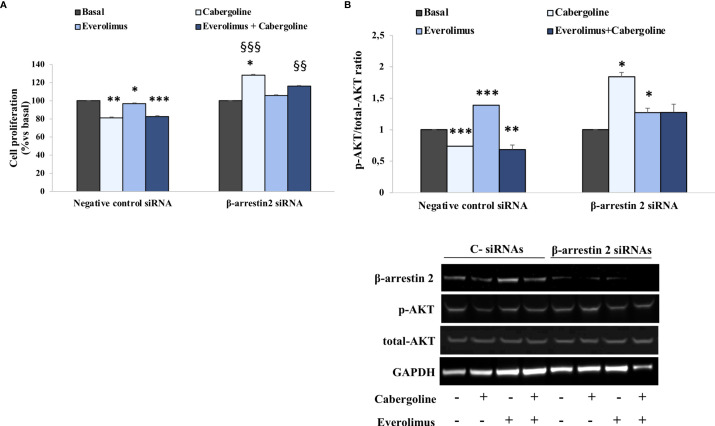
β-arrestin 2 lack reverted everolimus and cabergoline cotreatment ability in reducing both p-AKT and cell proliferation in MMQ cells. The MMQ cells were transiently transfected with β-arrestin 2 siRNAs or negative control siRNAs (C-) for 72 h and then incubated with 100 nM cabergoline and 1 nM everolimus for 72 h **(A)** or 3 h **(B)**, administered singularly or in combination. **(A)** BrdU incorporation in newly synthesized DNA was measured. The experiments were repeated 4 times, and each determination was done in quadruplicate. Values represent mean ± SD normalized *vs*. respective basal. *p < 0.05, ***p < 0.001 *vs*. corresponding basal, ^§§^ *p* < 0.01; ^§§§^ *p* < 0.001 *vs*. corresponding treated negative control. **(B)** Densitometrical analysis of p-AKT (Ser473)/total AKT ratio normalized *vs*. respective basal (mean ± SD from 3 independent experiments), and representative immunoblots are shown. The equal amount of proteins was confirmed by stripping and reprobing with an anti-GAPDH antibody. *p < 0.05, **p < 0.01, ***p < 0.001 *vs*. corresponding basal.

In order to test everolimus’ effects on AKT activity, the AKT phosphorylation levels on Ser473 were evaluated by western blot analysis after 3 h of incubation with everolimus and/or cabergoline. As shown in **Figure 3B**, everolimus significantly increased the p-AKT/total-AKT ratio (+1.53 ± 0.24-fold, *p* < 0.001 *vs*. basal), while cabergoline induced a reduction (-18.6 ± 5.6%, *p* < 0.001 *vs*. basal). Cotreatment with cabergoline and everolimus resulted in a strong decrease of p-AKT/total-AKT ratio (-34.5 ± 14%, *p* < 0.001 *vs*. basal) (**Figure 2B**).

### β-Arrestin 2 Silencing Prevented Everolimus and Cabergoline Cotreatment Inhibitory Effects on Both p-AKT and Cell Proliferation in MMQ Cells

To test a possible involvement of β-arrestin 2 in mediating cabergoline’s inhibition of everolimus-induced AKT phosphorylation, genetic silencing experiments were performed in MMQ cells. Our data show that cell proliferation inhibition induced by cabergoline, both alone and in combination with everolimus, was reverted by β-arrestin 2 silencing (**Figure 3A**). Accordingly, the lack of β-arrestin 2 prevented the ability of cabergoline to reduce the p-AKT/total-AKT ratio after 3 h of exposure to everolimus (**Figure 3B**).

In addition, in cells silenced for β-arrestin 2, cabergoline induced a stimulatory effect on AKT according to the observed increase in cell proliferation (**Figures 3A, B** **)**.

## Discussion

This study demonstrated that the cotreatment with the mTOR inhibitor everolimus and DRD2 agonist cabergoline is able to overcome the resistance of a consistent subgroup of NF-PitNETs to everolimus’ antiproliferative effects by preventing the upstream activation of AKT.

This work’s results highlighted that about two-thirds of NF-PitNETs were resistant to everolimus treatment in terms of cell proliferation inhibition, and almost all tumors of this subgroup were also resistant to cabergoline. However, the coadministration of everolimus and cabergoline was able to significantly reduce cell proliferation, while it did not potentiate the effects of each agent singularly administered in responsive tumors.

In agreement, it has been previously shown that a subset of NF-PitNETs is resistant to the antiproliferative *in vitro* effects of everolimus (8, 11) or rapamycin (10). In particular, Zatelli and co-authors demonstrated that everolimus, used at a higher concentration compared to the present investigation, was effective in reducing cell proliferation in about 70% of NF-PitNETs, and cabergoline did not potentiate the effect of everolimus in responsive tumors (8). Moreover, a study by Rubinfeld *et al.* unveiled that the limited tumoral responsiveness to mTOR inhibitors in human-derived NF-PitNET cells could be overtaken by combining different kinds of drug or targeting multiple players of the PI3K–AKT–mTOR pathway, emphasizing the cell type-specific effects of these treatments (11). Another work suggested that mTOR inhibitors’ efficacy could be improved by cotreatment with other drugs. Particularly, it was shown that, in NF-PitNETs, octreotide cotreatment potentiated the effects of rapamycin and conferred responsiveness to resistant NF-PitNETs (10).

A lot of potential mechanisms leading to mTOR inhibitor resistance have been described in literature in various kinds of tumors (12)—for instance, earlier studies suggested that tumors with PTEN, FKBP-12, or FKB domain mutations or constitutive PI3K activity displayed scarce responses to mTOR inhibitors. Alterations in protein translation (decreased 4E-BP1 or increased eIF4E) have also been demonstrated to interfere with the effects of mTOR inhibitors on protein synthesis. The stimulation of autophagy and the increased levels of anti-apoptotic molecules, such as Bcl-2, represent additional mechanisms of resistance. Moreover, non-functional apoptotic pathways have been highlighted to potentially confer resistance together with modulation of apoptotic regulators’ stimulation of autophagy and enhanced angiogenesis. In addition, another way can be addressed to the increase in ERK/MAPK signaling, activation of the serin/threonine PIM kinases, the activation status of PDK1, or the altered expression levels of 4E-BP1, a downstream substrate of mTOR, which suppresses eIF4E activity. Furthermore, it was demonstrated that preventing the downregulation of p27Kip1 levels can lead to a less response to this kind of drugs (12). Nevertheless, the increase in AKT activation has been shown to be one of the major contributors that diminished everolimus’ anticancer activity effectiveness, which was induced by an escape mechanism.

Specifically, in different human-derived tumoral cell lines, everolimus blocked mTOR’s ability in inhibiting the ribosomal protein S6 kinase (p706SK), determining an activation of IRS-1 that leads to AKT phosphorylation. This cascade of events flows into an increase of AKT activity and, consequently, into an enhancement of cell proliferation (13).

This suggests that a combined therapy that inhibits mTOR function and prevents AKT activation might have improved the antitumoral activity.

A possible therapeutic potential of DRD2 as a target in PitNETs has been also reported in a previous study in which it was stated that, in ACTH-secreting PitNET cell model, an association of 9-cis retinoic acid and the DRD2 agonist bromocriptine modulates the receptor’s signaling in terms of hormone secretion and cell viability (25).

We have recently demonstrated that the DRD2 agonist cabergoline was effective in inhibiting AKT phosphorylation through a β-arrestin 2-dependent mechanism in tumoral lactotrophs and NF-PitNETs (21). Here we showed that cabergoline improved everolimus’ efficacy by blocking the AKT upstream reactivation.

The AKT phosphorylation levels were analyzed in order to study its activation status after everolimus and cabergoline treatment, singularly and in combination. Our data demonstrated that everolimus-unresponsive NF-PitNETs showed a substantial enhancement of the p-AKT/total-AKT ratio when incubated with everolimus alone, in agreement with the escape mechanism mentioned above. On the other side, the combined treatment with cabergoline strongly reduced this increase of AKT activity, and this ability is correlated with its antimitotic effect. In order to test the relevance of DRD2 expression for NF-PitNETs’ responsiveness to cabergoline and to evaluate the different expressions in DRD2 or its isoforms, long (D2L) and short (D2S), RT-PCR was performed. From our analysis, it emerged that all tumors positively expressed both isoforms of DRD2, regardless of responsiveness to dopamine agonist, ruling out a possible a role of both specific DRD2 isoforms in mediating opposite effects on the everolimus escape mechanism. Our results are in accordance with the observation that the clinical sensitivity to DAs was not associated with the expression of DRD2 or its isoforms (26).

In this sense, we might assume that everolimus’ poor efficacy in NF-PitNETs might be caused by the loss of the negative upstream feedback on AKT, thereby determining the resistance to treatment.

An effect of cabergoline in potentiating the effect of everolimus administration by a reduction of the mTOR inhibitor-induced escape mechanisms was previously reported in lung carcinoid tumoral cells (27).

In PRL-secreting PitNET MMQ cells, cabergoline determined a significant reduction of both cell proliferation and the p-AKT/total-AKT ratio as previously reported (21). On the contrary, everolimus was unable to reduce cell proliferation due to a significant increase of AKT phosphorylation. This effect was reverted by the combined treatment with cabergoline. The co-incubation with everolimus and cabergoline significantly induced a reduction of both cell proliferation and the p-AKT/total-AKT ratio with respect to cabergoline treatment alone, suggesting a possible synergic effect. These observations allow us to consider MMQ cells as a model of “everolimus escape”. Conversely, in rat somatolactotroph tumoral GH3 cells that are responsive to everolimus’ antiproliferative effects, no increase of AKT activity was detected after the everolimus treatment (28). With regard to this, a previous study demonstrated that, in aggressive PRL-PitNETs, everolimus exhibited an antiproliferative action *in vitro*, suggesting it as a novel therapeutic option in PRL-PitNETs resistant to conventional therapy with cabergoline. Moreover, they demonstrated how the everolimus and cabergoline combination determined *in vivo* tumor size reduction and PRL level normalization (29).

In addition, we demonstrated the involvement of β-arrestin 2 in mediating cabergoline’s inhibition of AKT activity and cell proliferation after everolimus cotreatment.

Genetic silencing experiments in MMQ cells revealed that the lack of β-arrestin 2 reverted the antimitotic effect induced by the combined treatment with everolimus and cabergoline as well as the inhibition of AKT. It should be noted that MMQ cells only express D2L (21, 30), and studies in literature seem to attribute the postsynaptic phenomena to D2L, such as AKT regulation (31).

However, a specific role of DRD2 isoforms in β-arrestin 2 recruitment has not yet been specifically investigated.

In conclusion, the present study revealed that cabergoline might overcome the everolimus escape mechanism in NF-PitNETs and tumoral lactotrophs by inhibiting the upstream AKT activation. The coadministration of cabergoline might improve mTOR inhibitors’ antitumoral activity, paving the way for a potential combined therapy in β-arrestin 2-expressing NF-PitNETs or other PitNETs resistant to conventional treatments.

## Data Availability Statement

The raw data supporting the conclusions of this article will be made available by the authors without undue reservation.

## Author Contributions

FM: conceptualization, methodology, investigation, data curation, writing—original draft, writing—review and editing, and formal analysis. EE, DT, RC, GM, GDM, and AMB: investigation. ML and AL: resources, review, and editing; AM, AS, and MA: review and editing. EP: conceptualization, validation, data curation, funding acquisition, supervision, writing—original draft, writing—review and editing, project administration, and formal analysis. GM: conceptualization, supervision, funding acquisition, project administration, and writing—review and editing.

## Funding

This work was supported by Associazione Italiana Ricerca Cancro grant to GM (IG 2017-20594), Italian Ministry of Health grant to GM (PE-2016-02361797), and Ricerca Corrente Funds from the Italian Ministry of Health and Progetti di Ricerca di Interesse Nazionale grant to EP (2017N8CK4K).

## Conflict of Interest

The authors declare that the research was conducted in the absence of any commercial or financial relationships that could be construed as a potential conflict of interest.

## Publisher’s Note

All claims expressed in this article are solely those of the authors and do not necessarily represent those of their affiliated organizations, or those of the publisher, the editors and the reviewers. Any product that may be evaluated in this article, or claim that may be made by its manufacturer, is not guaranteed or endorsed by the publisher.
