# Supplementary figures and images for: DRD2 Agonist Cabergoline Abolished the Escape Mechanism Induced by mTOR Inhibitor Everolimus in Tumoral Pituitary Cells
Source: Front Endocrinol (Lausanne). 2022 Jun 3;13:867822. doi: 10.3389/fendo.2022.867822 (PMC9204243; doi:10.3389/fendo.2022.867822)

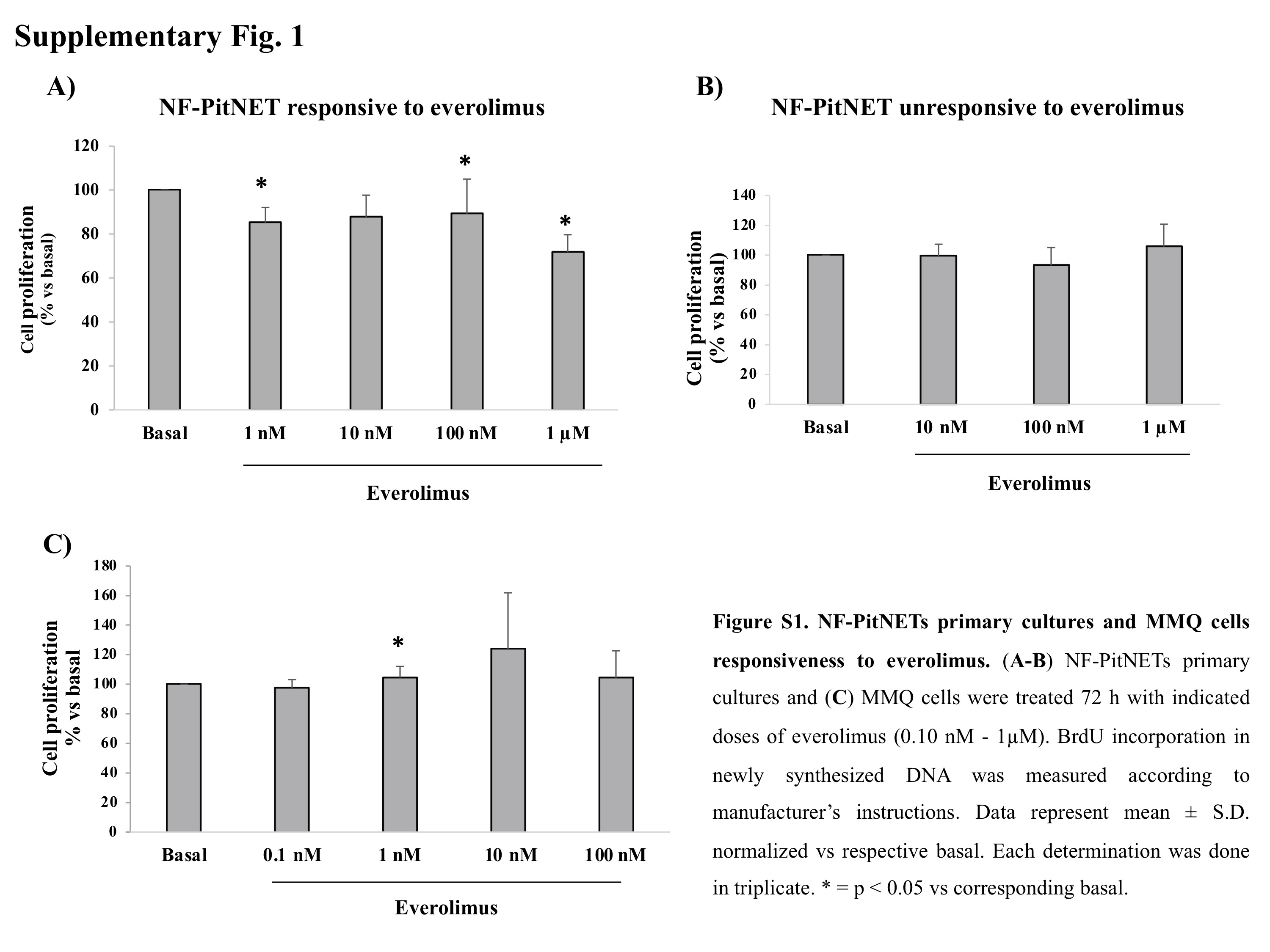

Supplement: Supplementary file 1 [file Image_1.jpg]
